# Real-time system for studies of the effects of acoustic feedback on animal vocalizations

**DOI:** 10.3389/fncir.2012.00111

**Published:** 2013-01-07

**Authors:** Mike Skocik, Alexay Kozhevnikov

**Affiliations:** ^1^Department of Physics, Pennsylvania State UniversityUniversity Park, PA, USA; ^2^Department of Psychology, Pennsylvania State UniversityUniversity Park, PA, USA

**Keywords:** acoustic feedback, animal vocalizations, behavioral neuroscience, sensory feedback, real-time data processing

## Abstract

Studies of behavioral and neural responses to distorted auditory feedback (DAF) can help shed light on the neural mechanisms of animal vocalizations. We describe an apparatus for generating real-time acoustic feedback. The system can very rapidly detect acoustic features in a song and output acoustic signals if the detected features match the desired acoustic template. The system uses spectrogram-based detection of acoustic elements. It is low-cost and can be programmed for a variety of behavioral experiments requiring acoustic feedback or neural stimulation. We use the system to study the effects of acoustic feedback on birds' vocalizations and demonstrate that such an acoustic feedback can cause both immediate and long-term changes to birds' songs.

## Introduction

Distorted auditory feedback (DAF) is used for assessing the effects of auditory input on vocal production. Presenting DAF and assessing its effects on the song and on the neural activity have been used in songbirds to study the mechanisms of song production and learning (Leonardo and Konishi, 1999; Sakata and Brainard, 2006; Andalman and Fee, 2009; Keller and Hahnloser, 2009; Tschida and Mooney, 2012). Human speech is sensitive to certain types of DAF (Lee, 1950; Houde and Jordan, 1998), and DAF is used to study speech mechanisms. It is often desirable to have real-time DAF, i.e., to rapidly (in a few milliseconds or faster) detect the occurrence of specific acoustic elements in vocalization and present an auditory stimulus once the target acoustic element is detected.

In this paper, we describe an automated system for real-time DAF and demonstrate its use to study both the immediate and the long-term effects of DAF on the song of Bengalese finches. The system uses open-source software and, therefore, is extremely flexible and customizable by the user. It has a significantly lower cost than commercial systems.

Songbirds use auditory feedback to learn to sing when they are young and to maintain their songs in adulthood (Konishi, 1965; Brainard and Doupe, 2000). Long-term exposure to DAF has been shown to cause song degradation in songbirds (Okanoya and Yamaguchi, 1997; Woolley and Rubel, 1997; Leonardo and Konishi, 1999). Some bird species' songs exhibit immediate sensitivity to acoustic input. For these birds, DAF can have an immediate effect on the timing and acoustic structure of the song (Sakata and Brainard, 2006). Analyzing the effects of DAF can yield new understanding of the neural organization of the song and the mechanisms of song learning (Brainard and Doupe, 2000). To study the questions about the effects of time-localized DAF on birdsong, it is important to be able to deliver DAF with high temporal precision in relation to vocalization. To do this, it is necessary to rapidly and reliably detect the specific acoustic elements of the bird's song and, after detection of the acoustic element, generate an acoustic output.

It is a challenging technical task for an acoustic feedback system to be real-time. Real-time performance is most easily achieved with analog systems (Cynx and Von Rad, 2001), but digital systems offer significant advantages in terms of convenience and flexibility. However, the advantages of a digital system are accompanied by the difficulties of making a digital system have small and constant processing delays. The system has to perform analog-to-digital conversion, fast analysis of the recently acquired data and digital-to-analog conversion, and these operations have to take place with reliable timing and concurrently with saving the acquired data. Custom-made DAF systems have been developed and used in behavioral studies (Leonardo and Konishi, 1999; Kao et al., 2005), but their real-time processing characteristics have not been reported. Oftentimes, custom-made systems have significant and not well-controlled delays, especially for systems based on PC's running Windows. Commercial systems for real-time acoustic processing are available but are expensive.

We developed a real-time DAF system based on a PC running Linux and the Real-Time eXperiment Interface (RTXI) software (Lin et al., 2010) and a National Instruments A/D card. The system is low-cost (the cost is only the cost of the hardware, the software is free). The system is capable of A/D bandwidth of over 30 kHz with real-time processing of acoustic signals.

## Methods

A PC with an Intel i7 six-core processor (2.66 GHz) and 4 GB or RAM running Ubuntu Linux 2.6.29.4-rtai with RTXI version 1.1.2 and a National Instruments PCIe-6251 A/D card is used. The A/D card receives audio input from a microphone (AudioTechnika PRO-44, used with Behringer Shark DSP110 microphone amplifier). The output is sent to a speaker amplifier (SLA-1, Applied Research and Technology); the output of the amplifier is connected to a speaker.

**Figure 1:**
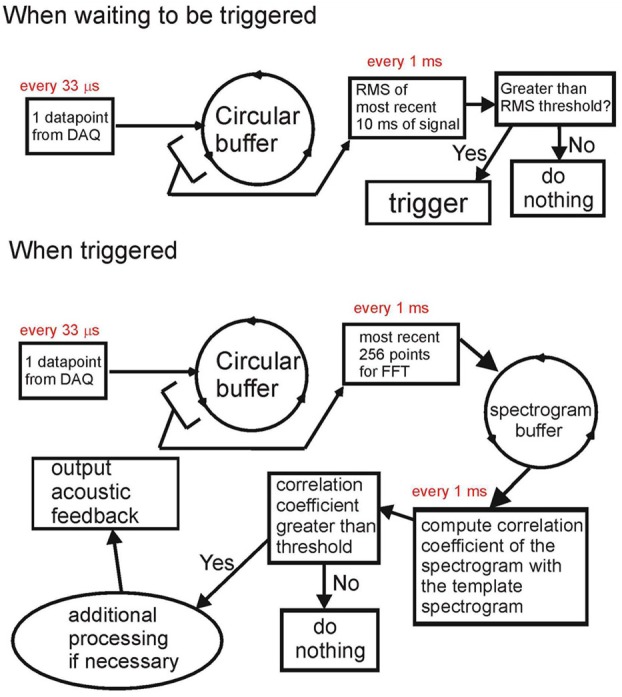
**Block diagram of the acoustic feedback system.** When not triggered **(top)**, the system computes the rms of the input signal. When the rms exceeds the threshold, the system is triggered. When triggered **(bottom)**, the system computes the spectrogram of the most recent 20 ms of signal and computes the correlation coefficient of this spectrogram with the spectrogram of the template sound (e.g., song syllable). The template sound is detected when the correlation coefficient exceeds a threshold value; in this case, acoustic feedback can be generated. Both the input and the acoustic output are saved to the computer hard drive.

The Data Recorder software within RTXI is custom modified. The simplified diagram of signal processing is shown in Figure 1. The system has two modes of operation—a non-triggered (idle) mode and a triggered (active) mode. At the core of the modified software is the circular buffer that takes data points one-by-one from the data acquisition engine once they become available. In the non-triggered mode [Figure 1 **(top)**], the system continuously (every 1 ms) computes the rms of the last 10 ms of the input signal. If the signal rms exceeds the threshold, the system is switched into triggered mode.

In triggered mode, the system does real-time processing of auditory data. In Figure 1 **(bottom)**, we show the processing done for recognizing the song syllable of a Bengalese finch. The FFT of the past 256 data points (~8.4 ms) is computed every 1 ms and stored in an FFT circular buffer. Every 1 ms, the spectrogram of the most recent 40 ms of the input signal is obtained from the FFT circular buffer. A correlation coefficient between the input signal spectrogram and the spectrogram of the template is computed. If the correlation coefficient exceeds the threshold, the system detects the occurrence of the target song syllable, and acoustic feedback can be generated, or further processing can be done. While generating acoustic feedback, the system keeps going through all of the above steps, but is disallowed from registering another detection to prevent it from triggering on its own output.

The presence of the data circular buffer allows very fast access to chunks of the most recent data for processing without affecting the timing of the data acquisition process. The FFT circular buffer also allows extremely fast computations of the spectrograms of the sound (computing the spectrogram is a computationally-intensive task). This enables the system to recognize complex vocal elements based on their spectrogram (e.g., frequency sweeps) without compromising the timing. While triggered, every 1 s, the system computes the rms of the previous 200 ms of the input signal to check if the acoustic input is still present. If the rms is below a threshold (no signal), the system goes into the idle mode. While triggered, the system continually saves all the data acquired in a separate array and saves the data to the hard drive once it is switched back to idle mode. A more detailed description of this system, along with the source code, is available at http://www.phys.psu.edu/~akozhevn/ac_feedback/.

## Results

We tested the performance of our DAF system in several tasks which are often needed in behavioral experiments using acoustic feedback. We also used the system to assess the effects of acoustic feedback on the song of Bengalese finch. All animal procedures were carried out in accordance with the locally approved IACUC protocol.

### Delay between input and output test

A simple task is generating acoustic feedback when the input level exceeds a certain threshold. Although this task may be too simple for most behavioral experiments, the delay in the system between detecting the crossing of the threshold and producing the output is a useful figure for indicating how fast the system can be when it is solely converting A/D and D/A and saving data without any complex data processing.

The system was programmed so that, once the input exceeded a fixed threshold, the acquired input signal was sent to the D/A output with no extra processing. A square wave with the amplitude exceeding the threshold was applied to the input; the delay between the input and the output was measured with the digital oscilloscope. The measured delay between the output and the input was 27 ± 9 μs (mean ± SD, min = 9 μs, max = 43 μs). The sample rate was 30.3 kHz, so the observed delays corresponded to a delay of 1 data point between the input and the output. The observed variations of the delay are due to the difference in timing between the external input signal and the timing of the A/D events. In all cases, however, the delay between the input and the output does not exceed 1 datapoint. Therefore, the system has real-time capability.

### Detection of specific vocal elements in the bird's song

A typical task in experiments using acoustic feedback is detection of a certain “template” sound. The template can be either a sound of a certain frequency or a more complex combination of frequencies, frequency sweeps, etc. Once the template is detected, the acoustic feedback can be played back to the animal. This task is computationally intensive because one needs to compute the characteristics of the recently acquired input signal, then compare these with the characteristics of the template and, if the input is sufficiently similar to the template, decide that the detection has occurred and generate acoustic output. The computation has to be done fast enough to enable real-time performance and not interfere with the data acquisition process.

Common techniques that have been used for detecting acoustic elements are spectrogram-based techniques (Leonardo and Fee, 2005) and feature-based techniques (Tchernichovski et al., 2000). In a spectrogram-based approach, the spectrogram of the recently acquired signal is computed and compared to the template spectrogram. A common way to accomplish this is to compute the correlation coefficient between the two spectrograms. Detection of the template sound occurs if the correlation coefficient exceeds a threshold value.

**Figure 2:**
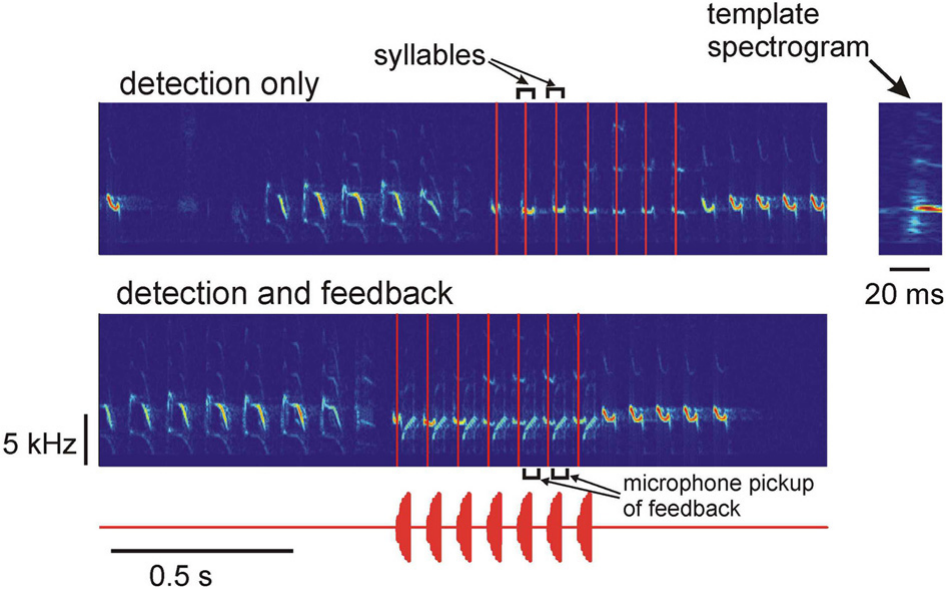
**Top:** spectrogram of the song of a Bengalese finch and the times of occurrence of one of the song syllables. The system was programmed to only detect the occurrences of the target syllable in real time, no acoustic feedback was generated. The detection times are shown as vertical red lines. **Bottom:** the system is detecting the target syllables (vertical red lines) and is generating acoustic feedback after detection. The acoustic feedback waveform is shown below. The feedback signal is one of the birdsong syllables; the acoustic feedback pickup by the microphone is visible on the spectrogram. The zoomed-in spectrogram of the template is shown on the right.

We tested the performance of the system for detection of specific syllables in the song of a Bengalese finch. The Bengalese finch song consists of a sequence of syllables separated by silences (inter-syllable gaps) (Figure 2). The acoustic structure of the song syllables is fairly stable; the main source of variability from one song to another is the sequence of syllables in each song (Honda and Okanoya, 1999).

To detect a specific song syllable, the system continuously computes the correlation coefficient of the spectrogram of the most recent 20 ms segment of the acquired signal with the spectrogram of a 20-ms syllable template (see Methods, Figure 1). The target syllable is detected by the system when the correlation coefficient exceeds the threshold value of 0.8. The value of the threshold was chosen by examining the target syllable detections by the DAF system in a set of about 20 songs and comparing the detected syllable occurrences with the actual occurrences of the target syllables determined by visual examination of the song spectrograms. If the threshold is set too high, the probability of missing the target syllable increases. Setting the threshold too low increases the probability of false positive detections. After the syllable detection, acoustic feedback (either white noise or the song syllable) can be played back to the bird.

Typical performance of the system on the real-time syllable recognition task is shown in Figure 2. The top spectrogram shows “detection only” mode—the system detects the target syllable in real time, but no playback is generated. The bottom spectrogram shows detection and playback generation—after detecting the target syllable, the system plays back another song syllable to the bird. The vertical red lines indicate the detection times of the target syllable. The zoomed-in spectrogram of the template is shown on the right. The template contains part of the inter-syllable interval and the first 20 ms of the target syllable, so the end of the template (detection time) is approximately in the middle of the 40-ms long syllable.

Performance of the system was checked by comparing the results of automatic detections of the system with the manual identification of the target song syllables carried out by off-line examination of the spectrograms. Out of 659 target syllables, 610 were correctly detected and 49 were missed. There were zero false positives. Thus, the system shows robust performance with the real-time syllable recognition task: over 92% of the target syllables were correctly identified.

This demonstrates that the system is capable of real-time detection of target syllables in the song. Note that the syllables occurring after the target syllables in Figure 2 are frequency sweeps that overlap with the template's frequencies. The system discriminates them from the target syllables because they have a different frequency profile. Such discrimination is an advantage of the spectrogram-based detection; this would not be possible if only instantaneous frequencies were detected.

Additionally, we tested the system on the detection of syllables in the song of a zebra finch—another bird species. We used our dataset of zebra finch songs with known syllable sequences obtained in a previous study (Kozhevnikov and Fee, 2007). Zebra finch songs were played back through the speaker, and the results of the real-time detection by our DAF system were compared to the known occurrences of the target syllable.

A small subset of songs (10 songs) was used as a test set: the threshold value for the syllable detection was adjusted to optimize the percentage of correctly detected syllables in this small test set. After this, the threshold kept was fixed, and the performance of the system was tested on the whole dataset (about 100 songs). Out of 756 target syllables in the dataset, 728 were correctly detected, 28 were missed; there were 4 false positives. The system correctly detected over 96% of the target syllables in the dataset; the probability of a false positive detection was less than 1%.

### Effects of auditory feedback on the timing of the birdsong

Auditory feedback has been shown to have immediate effects on some animal vocalizations. For Bengalese finches, DAF has been shown to affect the timing of song syllables. DAF played after the song syllable increases the time interval between that syllable and the next syllable in the song (Sakata and Brainard, 2006). We tested whether our feedback system is effective in causing real-time changes to the Bengalese finch song. The system was programmed to detect one of the song syllables and, once the syllable was detected, to play back the same song syllable with a probability of 0.05. This ensured that the feedback was sufficiently sparse so almost in all cases there was only one playback during each song. The feedback and control trials were randomly interleaved. This simplified the analysis of the syllable timing and eliminated any confounding effects from playbacks being too close to one another. The delay between the syllable sung by the bird and the syllable playback was 40 ms.

The playback causes some pickup on the input channel, which can cause difficulties in precise determination of the timing of the syllable that is occurring during the playback. Therefore, the time interval between the target syllable and the following syllable (which is partially overlapped with playback) was computed as one half of the difference between the detection time of the target syllable and the detection time of the second syllable after the target syllable. The same procedure was performed in control trials to ensure consistency in data analysis. Since the distributions of time intervals may not be Gaussian, we use a non-parametric statistical test—two-way Kolmogorov–Smirnov test—to assess the statistical significance of DAF effects on the song timing.

**Figure 3:**
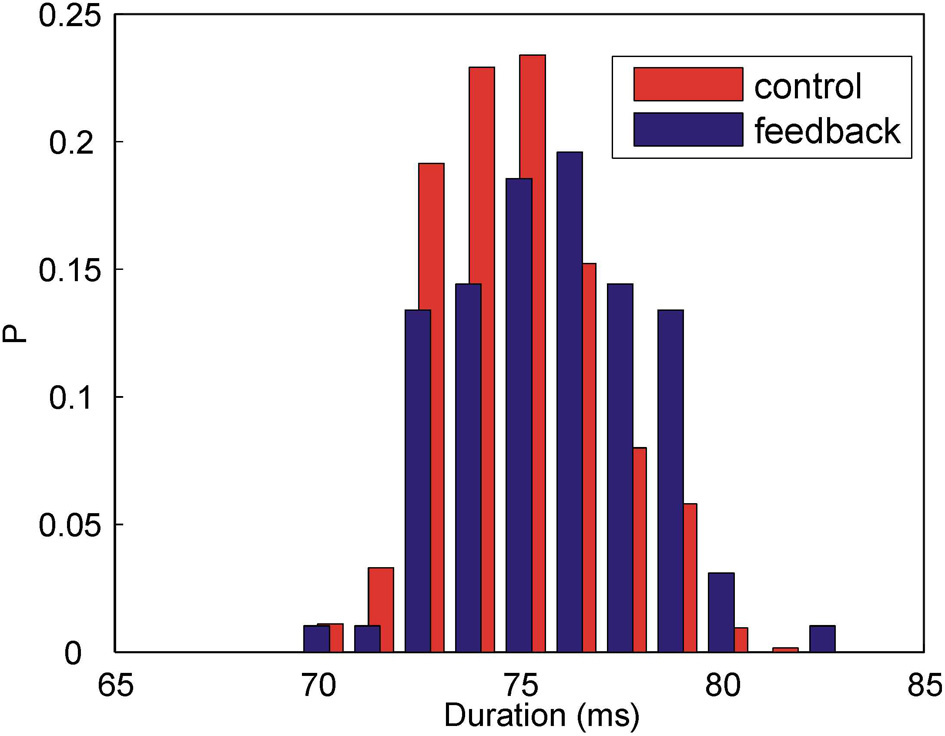
**DAF increases the duration of the time interval between Bengalese finch song syllables.** Shown above are the histograms of the time intervals between two subsequent syllables in the song in the presence of DAF (blue) and without DAF (red). The means are: Δ *t*_mean_ = 74.8 ms (control, *N* = 637 syllables) and Δ *t*_mean_ = 75.7 ms (feedback, *N* = 97 syllables), the difference is statistically significant (*p* = 0.001, two-way Kolmogorov–Smirnov test).

Figure 3 shows the distributions of the time intervals between the target syllable and the following syllable when the feedback is present (blue histogram) and when there is no feedback (red histogram). The widths of the distributions are due to the natural variability of the song timing. In the presence of feedback, the time intervals between the syllables become longer. Without DAF, the mean interval is 74.8 ms (*N* = 637 syllables); in the presence of DAF, the mean interval is 75.7 ms (*N* = 97 syllables). Although the change of the mean duration is small compared to the widths of the distributions, the effect is highly statistically significant (*p* = 0.001, two-way Kolmogorov–Smirnov test). The observed lengthening of the time interval between the song syllables is consistent with previous observations (Sakata and Brainard, 2006). Thus, our acoustic feedback has an immediate effect on the song: DAF immediately and reversibly affects song timing.

### Long-term effects of daf on acoustic structure of the song

DAF has been shown to cause long-term changes to animal vocalizations. For songbirds, prolonged repeated presentation of DAF can cause gradual change of the song (Leonardo and Konishi, 1999; Warren et al., 2011). We tested out system on the task of causing long-term changes of the frequency of one of the song syllables.

The system is programmed to detect the fundamental frequency of one of the song syllables. After the target song syllable is detected, the temporal profile of the pitch (defined as the largest peak in the FFT of the latest 256 points) was computed. The lowest value in the pitch profile in the time window between 3 and 12 ms after the detection time was taken to be the pitch of the syllable. The feedback (white noise) is conditional on the detected pitch of the song syllable. For example, the feedback can be generated if the detected syllable pitch is smaller than a threshold value. Continuous exposure to such feedback has been shown to cause the bird to gradually shift the mean pitch of the syllable so that the feedback is generated less often—the bird adapts its song to avoid hearing DAF (Warren et al., 2011).

**Figure 4:**
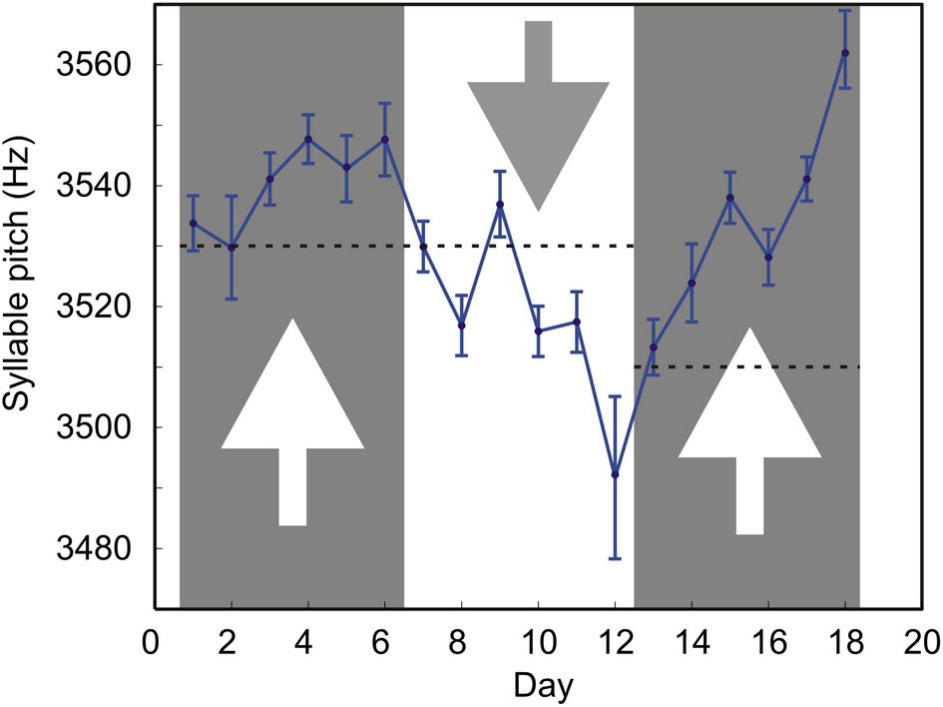
**Prolonged exposure of the bird to DAF causes gradual changes in the song.** The mean pitch of one of the target song syllable was manipulated by feedback conditional on pitch. Each datapoint is the pitch of the target syllable averaged over all the renditions of the target syllable sung by the bird on a given day. The number of renditions varies from day to day (mean = 208, min = 126, max = 288). Error bars are s.e.m. Dashed lines are the values of the threshold. Arrows indicate the direction in which the syllable frequency was expected to change in response to DAF. Larger error bars on day 12 are mostly due to the smallest number of target syllable renditions (*n* = 126) sung on that day.

We tested whether such conditional DAF could shift the mean syllable pitch in both directions. Figure 4 shows the long-term effects of DAF which is conditional on the syllable pitch. During days 1–6, the feedback was played back if the pitch was less than 3530 Hz; during days 7–12, the feedback was played back if the pitch was greater than 3530 Hz; during days 13–18, the feedback was played back if the pitch was less than 3510 Hz.

Our feedback is effective in causing gradual changes in the syllable pitch. Playing back DAF when the frequency is lower than the threshold value causes an upward drift of the mean pitch (days 1–6 and 13–18). Presenting feedback when the pitch is higher than the threshold causes a downward drift in pitch (days 7–12). This shows that the system is suitable for studies of long-term effects of DAF on animal vocalizations.

## Discussion

The described real-time acoustic feedback system is a versatile tool for studies of the effects of auditory feedback on ongoing animal vocalization. The advantage of the system is the flexibility of the processing than can be realized. The circular buffer allows real-time acquisition of arbitrary-length segments of the most recent data without affecting the timing of data acquisition. In addition, having a separate FFT buffer facilitates real-time spectral processing of acquired signals. This feature enables a very quick creation of the spectrograms of long (tens or even hundreds of milliseconds) segments of signals at a high rate (the spectrogram is updated every 1 ms).

This capability is very useful for the detection of complex vocal signals. Often, it is not just a certain frequency that needs to be detected, but rather a certain spectrogram pattern, like multiple frequencies or the frequency sweeps frequently seen in birdsong syllables. Since the same frequency can occur in many syllables, it is the whole pattern of the spectrogram that allows real-time detection of the syllable. Our system is very well-suited for rapid spectrogram-based detection of acoustic elements.

The performance of the system will vary depending on the type of animal vocalization and the nature of the acoustic element being detected for two main reasons. First, there is always a natural variability in the acoustic structure of a vocal element, and the degree of this variability may be different for different vocal elements; this will affect the reliability of detection. For example, a birdsong syllable can possess a more or less stereotyped spectrogram; the detection will be easier for a more stereotypical song syllable. Second, a given vocal element can be more or less similar in its acoustic structure to other vocal elements; reliable detection of a target vocal element will be easier if it is spectrally more dissimilar to other vocal elements. To achieve optimum performance, adjustments to the threshold or detection algorithm may be needed; thus, it is important to have a highly customizable system.

It is worth mentioning that, when the song syllables are detected, data processing is not a time-limiting step, and significantly more complex processing can be done without decreasing the A/D rate. We tested the system with longer templates (60 and 100 ms); they did not affect performance. We also tested the simultaneous detection of two templates, so that, every 1 ms, the system computed the correlation coefficient of the sound spectrogram with two template spectrograms, and that also did not affect the A/D rate. For a template 60 ms long, the computation of the correlation coefficient takes 16 μs; this time scales linearly with the length of the template. The computation of the FFT (to fill the column in the spectrogram buffer) takes 7–8 μs. FFT and correlation coefficient computations are the slowest signal processing steps; all other steps combined take less than 1 μs. Thus—If the spectrogram update rate is kept at 1 ms—the system should be capable of simultaneously detecting of over 10 different song syllables. Therefore, fairly complex real-time analysis and detection of multiple vocal elements can be done without compromising the speed of the system.

The system is usually used with 1 output channel and 2 input channels (one input channel for acoustic input and one channel for recording the actual output of the A/D card). It is possible to increase the number of input channels. This way, one could use the data recording capability of the system to collect physiological data (e.g., EEG or neural) during acoustic feedback experiments. However, the process limiting the speed of the system appears to be reading the data from the A/D card and sending the data to the D/A. Thus, increasing the number of channels will slow the system down and decrease the A/D rate. We tested the performance of the system with 3 input channels and 1 output channel. To achieve stable operation, the A/D rate had to be decreased to 20 kHz. Despite this decrease, this is an acceptable rate for many experiments where acoustic and electrophysiological data have to be collected.

Finally, the real-time processing capabilities of the system could be used for neural feedback experiments. The spectrogram-based signal processing capability can be useful for the detection of neural oscillations. The output can be used for targeted microstimulation. The described auditory feedback system is a flexible low-cost tool for behavioral neuroscience research.

## Conflict of interest statement

The authors declare that the research was conducted in the absence of any commercial or financial relationships that could be construed as a potential conflict of interest.
